# Translational Potential of Fluorescence Polarization for Breast Cancer Cytopathology

**DOI:** 10.3390/cancers15051501

**Published:** 2023-02-27

**Authors:** Peter R. Jermain, Dina H. Kandil, Alona Muzikansky, Ashraf Khan, Anna N. Yaroslavsky

**Affiliations:** 1 Advanced Biophotonics Laboratory, University of Massachusetts Lowell, Lowell, MA 01854, USA; 2 Department of Radiation Oncology, Massachusetts General Hospital, Boston, MA 02114, USA; 3 Department of Pathology, UMass Chan Medical School, Worcester, MA 01655, USA; 4 Biostatistics Center, Massachusetts General Hospital, Boston, MA 02114, USA; 5 Department of Pathology, UMass Chan Medical School-Baystate, Baystate Health, Springfield, MA 01199, USA; 6 Department of Dermatology, Massachusetts General Hospital, Boston, MA 02114, USA

**Keywords:** breast cancer, cytopathology, methylene blue, fluorescence polarization, microscopy

## Abstract

**Simple Summary:**

The evaluation of breast fine needle aspiration cytology specimens requires subjective, visual assessments of cytomorphology, resulting in suboptimal diagnostic accuracy. The fluorescence polarization of methylene blue demonstrated significant potential as a quantitative marker for cellular level breast cancer diagnosis in clinical aspirates. Results indicate the technology could be implemented as a standalone approach for breast cancer detection in singe cells or augment conventional approaches to reduce the incidence of indeterminate cytopathology.

**Abstract:**

Breast cancer is the most common malignancy in women. The standard of care for diagnosis involves invasive core needle biopsy followed by time-consuming histopathological evaluation. A rapid, accurate, and minimally invasive method to diagnose breast cancer would be invaluable. Therefore, this clinical study investigated the fluorescence polarization (*Fpol*) of the cytological stain methylene blue (MB) for the quantitative detection of breast cancer in fine needle aspiration (FNA) specimens. Cancerous, benign, and normal cells were aspirated from excess breast tissues immediately following surgery. The cells were stained in aqueous MB solution (0.05 mg/mL) and imaged using multimodal confocal microscopy. The system provided MB *Fpol* and fluorescence emission images of the cells. Results from optical imaging were compared to clinical histopathology. In total, we imaged and analyzed 3808 cells from 44 breast FNAs. *Fpol* images displayed quantitative contrast between cancerous and noncancerous cells, whereas fluorescence emission images showed the morphological features comparable to cytology. Statistical analysis demonstrated that MB *Fpol* is significantly higher (*p* < 0.0001) in malignant vs. benign/normal cells. It also revealed a correlation between MB *Fpol* values and tumor grade. The results indicate that MB *Fpol* could provide a reliable, quantitative diagnostic marker for breast cancer at the cellular level.

## 1. Introduction

Breast cancer is the second leading cause of cancer-related deaths and the most frequently diagnosed cancer in women in the United States [1]. Its incidence has increased considerably over the past few decades [1,2]. When cancer is suspected, tissue is obtained from a core-needle or surgical biopsy and processed for histopathology. Diagnosis is delivered by pathologists following the examination of histological slides under a light microscope. The analysis is subjective and suffers from variations in interpretation, especially in borderline lesions [3,4]. Moreover, biopsy preparation for pathologic examination requires extensive, lengthy, and labor-intensive tissue processing. As some benign cells and lesions can closely mimic breast carcinoma, immunohistochemistry (IHC) staining techniques may be required to identify cancer-specific markers. However, the widespread clinical use of diagnostic ancillary markers is hindered by low throughput methods, prohibitively long turnaround times, and high costs [5].

Fine needle aspiration (FNA) cytology has been introduced as a more rapid, less invasive procedure that detects breast cancer at the cellular level. This approach is ideal for many palpable breast masses due to ease of access and overall trends towards minimally invasive procedures. However, due to the lack of tissue architecture, it relies solely on cytomorphological assessments of single cells. Hence, the diagnostic accuracy of FNA is 65.4%, which is significantly lower as compared to 88.7% for tissue histopathology [6]. Therefore, the improvement of the existing methods and development of novel approaches for the detection of breast cancer remain focal points in pathology and oncology research [7,8,9,10,11,12,13,14,15,16,17].

Recently, we proposed to address the problem of cellular-level cancer diagnosis by measuring the fluorescence polarization (*Fpol*) of cells stained in aqueous methylene blue (MB) [18,19,20,21]. Methylene blue is an ideal exogenous fluorophore for *Fpol* measurements in FNA specimens because it is a widely used cytological stain in pathology/oncology clinics and approved by the United States Food and Drug Administration for use in vivo [22,23,24,25,26].

*Fpol* measures the polarization state of light emitted by a specimen that was excited with linearly polarized light [27,28,29]. It is defined as *Fpol* = ($I_{//}-I_{⊥})/(I_{//}+I_{⊥}$), where $I_{//}$ and $I_{⊥\;}$ are fluorescence emissions polarized in the plane parallel and perpendicular to that of the incident light, respectively [27]. Previously, we successfully applied MB *Fpol* imaging for the delineation of breast tumor margins [30]. We also showed that MB *Fpol* is significantly higher in cultured breast cancer cells as compared to normal epithelial breast cells [18]. This study explored *Fpol* of MB as a quantitative marker for breast cancer in clinical FNA specimens.

## 2. Materials and Methods

### 2.1. Study Design

This clinical study evaluated multimodal confocal imaging for differentiation of malignant, benign, and normal breast FNA samples. Methylene blue *Fpol* images provided quantitative assessments of the cells, whereas MB fluorescence emission images yielded cytomorphological information. Aspirates were obtained from freshly excised, discarded breast tissues following partial or total mastectomy procedures at the University of Massachusetts Memorial Medical Center (UMMMC) in Worcester, Massachusetts. Any lesions less than 5 mm in greatest dimension were excluded. The specimens were of diverse histological classifications, tumor grades, and molecular subtypes. Cancerous samples included invasive ductal carcinoma (IDC) and invasive lobular carcinoma (ILC), whereas benign samples included fibroadenoma (FA) and intraductal papilloma (IDP). Multimodal optical images were acquired and *Fpol* values were measured in single cells. Results from optical imaging were compared against clinical histopathology. The study pathologists were blinded to all optical data and images. Therefore, standard patient diagnosis and treatment were not impacted in any way.

### 2.2. Sample Acquisition and Handling

Samples were collected from excess breast tissues by the study cytopathologist at UMMMC Surgical Pathology Laboratory. Each specimen was aspirated with a 22-gauge needle (Becton, Dickinson and Company, Franklin Lakes, NJ, USA) to provide material for optical imaging tests and cytology.

Aspirates for optical imaging were placed in 1.5 mL vials filled with Leibovitz’s L-15 medium (Sigma Aldrich, St. Louis, MO, USA) and transported at 37 °C within 1 h to the Advanced Biophotonics Laboratory (ABL) at the University of Massachusetts Lowell. At ABL, specimens were plated in glass bottom Petri dishes (In Vitro Scientific, Mountain View, CA, USA). The samples were put in an incubator (AR36L, Percival Scientific, Perry, IA, USA) maintained at 37 °C and 95% relative humidity for 12–24 h to allow cell attachment. Cell monolayers were stained for 20 min in MB solution (1% injection, McKesson Corporation, San Francisco, CA, USA) diluted to a concentration of 0.05 mg/mL using L-15 medium. After staining, cell layers were rinsed 3 times with 1X Phosphate Buffered Saline (PBS) (Fisher Scientific, Hampton, NH, USA) to remove surplus dye. Directly following the staining protocol, confocal images of the cells were acquired at the ambient temperature of 18 °C.

### 2.3. Confocal Imaging

**Figure 1:**
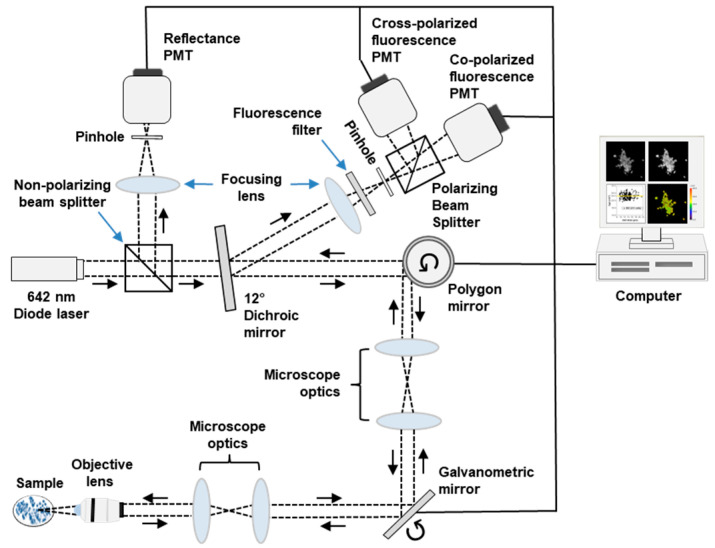
Schematic diagram of confocal imaging system. Dashed black line traces the optical path. PMT—photomultiplier tube.

Fluorescence images were acquired using the multimodal confocal microscope depicted in Figure 1. Full descriptions of the system are available elsewhere [18]. In brief, a 642 nm diode laser (Micro Laser Systems, Garden Grove, CA, USA) provided linearly polarized illumination. Images were acquired using a 63X/NA 1.4 oil immersion objective lens (Carl Zeiss, Oberkochen, Germany). Fluorescence emission was filtered using a 690 nm bandpass filter with full width at half maximum (FWHM) of 20 nm (Chroma, Bellows Falls, VT, USA) and focused by a lens onto a pinhole with 100 μm diameter (Edmund Optics, Barrington, NJ, USA). Fluorescence emission was separated into co- and cross-polarized components using a polarizing beam splitter (Karl Lambrecht Corporation, Chicago, IL, USA). The signals were detected concurrently by two photomultiplier tubes (PMT) (Hamamatsu Photonics, Shizuoka, Japan). Fluorescence signals were recorded as 8-bit grayscale images. The field of view was 205 µm × 205 µm. Lateral and axial resolution of the system were 0.9 μm and 3 μm, respectively.

### 2.4. System Calibration

The confocal microscope exhibits bias in the transmission of different polarization states of light. Therefore, to enable accurate measurement of *Fpol* values, we calculated the system calibration coefficient (*G*-factor) [27,31].

Two solutions of MB were prepared; one solution was 0.05 mg/mL MB dissolved in 1X PBS and the other was 0.05 mg/mL MB dissolved in glycerol (Fisher Scientific, Hampton, NH, USA). The MB dissolved easily in PBS, whereas the MB-glycerol solution was stirred for 20 min using a linear shaker (SK-0 330-Pro, Scilogex, Rocky Hill, CT, USA) to achieve uniform concentration. Two separate glass bottom Petri dishes were filled with 1.5 mL of the MB-PBS or MB-glycerol solutions.

The multimodal confocal microscope was used to acquire fluorescence images of each solution, with vertically or horizontally polarized excitation light (excitation wavelength: 642 nm), and by detecting vertically or horizontally polarized components of the fluorescence emission. Orientation of the excitation and emission polarizers was adjusted using a quarter-wave plate (Thorlabs, Inc., Newton, NJ, USA). Average intensity of each image was used to calculate *Fpol* of the solution, given by Equations (1) and (2):

(1) $$Fpol_{v}=\frac{I_{vv}-G\cdot I_{vh}}{I_{vv}+G\cdot I_{vh}}$$

(2) $$Fpol_{h}=\frac{I_{hh}-G^{-1}\cdot I_{hv}}{I_{hh}+G^{-1}\cdot I_{hv}}$$

where $Fpol_{v}$ and $Fpol_{h}$ represent *Fpol* values obtained with vertically or horizontally polarized excitation, respectively. Average fluorescence intensities are specified by $I_{vv}$ (vertically polarized excitation, vertically polarized emission), $I_{vh}$ (vertically polarized excitation, horizontally polarized emission), $I_{hh}$ (horizontally polarized excitation, horizontally polarized emission), and $I_{hv}$ (horizontally polarized excitation, vertically polarized emission).

*Fpol* is a property of the solution, and irrespective of the instrument used to measure it. Therefore, *Fpol* remains equal using vertically or horizontally polarized excitation ($Fpol_{v}$ = $Fpol_{h}$) and the G-factor can be calculated from Equation (3):

(3) $$G=\sqrt{\frac{I_{vv}\cdot I_{hv}}{I_{hh}\cdot I_{vh}}}$$

The *G*-factor was calculated to equal 0.75 independently for both solutions (MB-PBS and MB-glycerol). To verify the correct value of *G*, *Fpol* measurements of both MB solutions obtained from the confocal imaging system were verified against corresponding measurements from a commercial spectrofluorometer (FluoroMax-4, Horiba, Edison, NJ, USA).

### 2.5. Image Processing

Images were processed using MetaMorph software (Molecular Devices, Sunnyvale, CA, USA). Co- and cross-polarized fluorescence emission images were thresholded to remove noise and saturated pixels. Pixel values selected for low and high thresholds were 2 and 254, respectively. Cells were manually segmented, and average intensity of each region was used to calculate the *Fpol* value [27]:

(4) $$\mathrm{Fpol}=\frac{I_{//}-G\cdot I_{⊥}}{I_{//}+G\cdot I_{⊥}}$$

where $I_{//}$ and $I_{⊥}$ represent intensity in co-polarized and cross-polarized fluorescence emission images, respectively. *G* is the calibration factor.

*Fpol* images were generated using a MATLAB code (MathWorks, Natick, MA, USA). Co- and cross-polarized images were averaged and background was corrected. Fluorescence difference ($I_{Difference}=I_{//}-G\cdot I_{⊥}\;$) and emission ($I_{Emission}=I_{//}+G\cdot I_{⊥}\;$) images were processed and used to generate the *Fpol* image ($Fpol=\frac{I_{Difference}}{I_{Emission}}\cdot 100$). Pseudo-colors were applied to the *Fpol* image using ImageJ (available at http://rsb.info.nih.gov/ij/, accessed on 1 January 2023).

### 2.6. Statistical Analysis

Statistical analysis was performed using a linear mixed-effects model that accounted for fixed effects and random effects in the measurements [32]. Least squares estimates of mean *Fpol* and corresponding standard errors were obtained for 3 diagnostic groups (malignant: IDC and ILC cases; benign: FA and IDP cases; and normal: normal cases) and 5 histologic groups (IDC, ILC, FA, IDP, and normal). Significance of differences between the groups was assessed (*p* < 0.001 was considered significant).

Impact of the differences in MB *Fpol* values depending on tumor grade was statistically analyzed using data from malignant specimens. The cancer cells were organized into 3 tumor grade groups (grade 1, grade 2, grade 3) based on clinical findings and least squares estimates of means and standard errors in *Fpol* values were obtained. The significance of differences between tumor grades was assessed (*p* < 0.05 was considered significant).

### 2.7. Cytopathology and Histopathology

Permanent en face hematoxylin and eosin (H&E) histopathology sections were processed for each specimen. In addition, cytology slides were prepared using a modified Papanicolaou staining protocol. The slides were digitized using a Zeiss microscope (Axioscope, Carl Zeiss, Oberkochen, Germany). A 5X/NA0.13 air immersion objective (Carl Zeiss, Oberkochen, Germany) was used for histology, whereas a 60X/NA1.2 water immersion objective (Olympus Corporation, Shinjuku, Japan) was employed for cytology slides. Diagnosis of each specimen was obtained by a study pathologist from tissue histopathology following World Health Organization (WHO) classification criteria for tumors of the breast [33].

## 3. Results

### 3.1. Increased MB Fpol in Cancerous Breast FNAs

In total, we investigated 44 breast FNA specimens (3808 cells) collected from discarded breast tissues of 28 female subjects between 20 and 87 years old. A summary of the samples is presented in Table 1 (columns 1–3). Tumor sizes ranged between 0.6 and 10 cm (mean: 2.2 ± 1.8 cm). There were 19 malignant (1577 cells), 10 benign (910 cells), and 15 normal (1321 cells) specimens. The cancerous samples included 15 IDC (1335 cells) and 4 ILC (242 cells). The benign samples included six FA (632 cells) and four IDP (278 cells). Clinical evaluations showed that all malignant samples were heterogeneous, containing cancerous and noncancerous cells, whereas the benign and normal specimens did not contain any cancer cells. Detailed information for each specimen is provided in Supplementary Table S1, columns 1–6.

**Figure 2:**
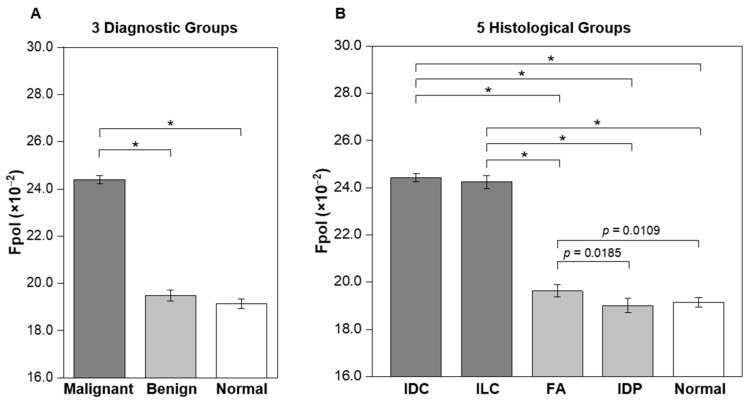
Average MB *Fpol* values of breast FNA specimens. (**A**) Three diagnostic groups including malignant (19 samples; 1577 cells), benign (10 samples; 910 cells), and normal (15 samples; 1321 cells). (**B**) Five histological groups including IDC (15 samples; 1335 cells), ILC (4 samples; 242 cells), FA (6 samples; 632 cells), IDP (4 samples; 278 cells), and normal (15 samples; 1321 cells). Error bars represent standard errors. * *p* < 0.0001.

Quantitative results, summarized in Table 1 (column 4) and Figure 2, demonstrate that the *Fpol* of MB is significantly higher in malignant breast FNAs vs. benign or normal. In Figure 2A, average *Fpol* for three diagnostic groups are shown (malignant: IDC and ILC cases; benign: FA and IDP cases; and normal: normal cases). The mean *Fpol* values for malignant, benign, and normal categories were 24.40 ± 0.17 (×10^−2^), 19.49 ± 0.23 (×10^−2^), and 19.14 ± 0.20 (×10^−2^), respectively. Differences between malignant vs. benign or normal groups were highly significant (*p* < 0.0001). Figure 2B presents average *Fpol* values for five histological groups (IDC, ILC, FA, IDP, and normal). The IDC and ILC groups exhibited average *Fpol* values of 24.42 ± 0.17 (×10^−2^) and 24.24 ± 0.27 (×10^−2^), respectively. The FA group had an average *Fpol* value of 19.63 ± 0.25 (×10^−2^), whereas that of the IDP group was 19.00 ± 0.30 (×10^−2^). There were significant differences (*p* < 0.0001) for each comparison of the cancerous vs. noncancerous groups. Furthermore, there were significant differences between FA vs. IDP (*p* = 0.0185), and FA vs. normal (*p* = 0.0109). Detailed *Fpol* data for all specimens are available in Supplementary Table S1, columns 7–10.

### 3.2. Quantitative Fpol Imaging of Breast FNA Specimens

**Figure 3:**
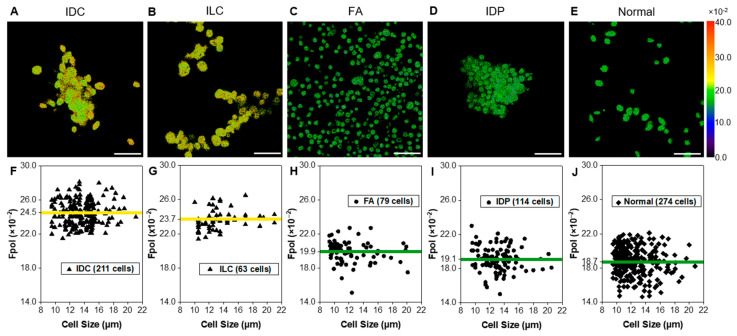
Quantitative assessment of representative breast FNA specimens. Example pseudo-colored MB *Fpol* images of cells including (**A**) IDC (subject 2-M), (**B**) ILC (subject 15-M), (**C**) FA (subject 23-B1), (**D**) IDP (subject 27-B), and (**E**) normal (subject 2-N). (**F**–**J**) Corresponding scatter plots of MB *Fpol* value (vertical axis) vs. cell size (horizontal axis) for all cells in the respective aspirates. Triangles—cells in malignant samples, circles—cells in benign samples, diamonds—cells in normal sample, yellow/green solid horizontal lines—average MB *Fpol* value of the sample. Scale bar = 50 µm.

Figure 3 presents quantitative *Fpol* data for five representative samples (one from each histological group). In the pseudo-colored *Fpol* images (Figure 3A–E), the color scale represents the *Fpol* value of each pixel, ranging from 0.0 to 40.0 (×10^−2^). Figure 3A shows example malignant cells (sample 2-M), obtained from moderately differentiated IDC, whereas Figure 3B displays cells (sample 15-M) aspirated from well differentiated ILC, classic type. Figure 3C,D display representative cells from FA (sample 23-B1) and IDP (sample 27-B), respectively. In Figure 3E, normal breast cells (sample 2-N) are shown. There is distinct contrast between the cells aspirated from malignant tumors (Figure 3A,B) (higher *Fpol* signals) relative to benign (Figure 3C,D) or normal cells (Figure 3E) (lower *Fpol* signals). In Figure 3F–J, scatter plots show *Fpol* values (vertical axis) vs. cell size (horizontal axis) for all cells in each representative sample and the average *Fpol* of the specimen (yellow/green solid horizontal lines). The malignant IDC (Figure 3F) and ILC (Figure 3G) samples had average *Fpol* values of 24.5 ± 1.4 (×10^−2^) and 23.7 ± 1.2 (×10^−2^), respectively. Average *Fpol* measured in the FA specimen (Figure 3H) was 19.9 ± 1.3 (×10^−2^), whereas in the papilloma case (Figure 3I) the average *Fpol* was 19.1 ± 1.4 (×10^−2^). The normal sample shown in Figure 3J had an *Fpol* value of 18.7 ± 1.7 (×10^−2^).

### 3.3. MB Fpol Scatter Plot of All Imaged Cells

**Figure 4:**
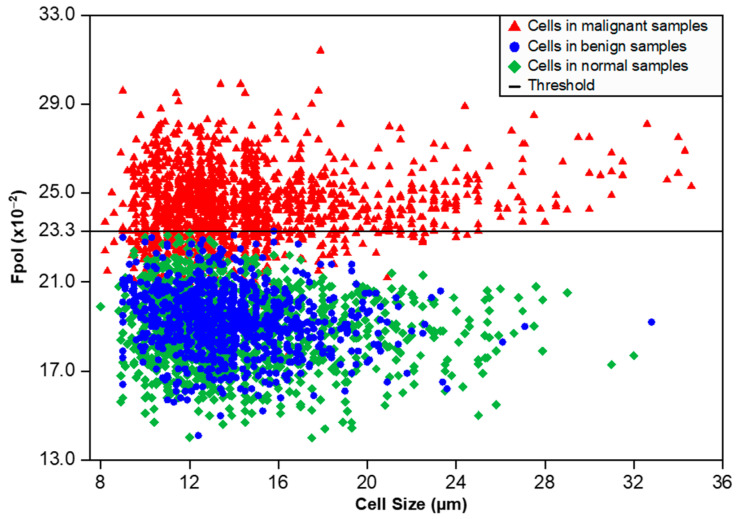
Scatter plot of MB *Fpol* value (vertical axis) vs. cell size (horizontal axis) for 3808 cells in 44 breast FNA specimens. Red triangles—cells in malignant samples (1577 cells), blue circles—cells in benign samples (910 cells), green diamonds—cells in normal samples (1321 cells), black solid horizontal line—MB *Fpol* value of 23.3 (×10^−2^).

Figure 4 displays a scatter plot of *Fpol* values vs. cell size for all 3808 cells investigated. Cells from benign samples (FA and IDP cases) and normal samples are shown as blue circles and green diamonds, respectively. Cells from cancerous samples (IDC and ILC cases) are represented by red triangles. Importantly, no cells in the noncancerous samples had an *Fpol* value above 23.3 (×10^−2^). Malignant cells, from FNAs characterized by various cancer types and/or disease progression, tended to exhibit higher *Fpol* values. The analysis of 19 cancerous specimens revealed that 1148 out of 1577 cells (73%) had *Fpol* above 23.3 (×10^−2^). Interestingly, 429 cells (27%) in cancerous aspirates presented MB *Fpol* less than or equal to 23.3 (×10^−2^). These lower values may be explained by the heterogeneity of the tumor specimens, as most cancerous aspirates contained some noncancerous cells (e.g., lymphocytes, etc.). It should also be noted that the threshold value, representing a cutoff criterion for malignancy in single cells, was empirically determined and may change as more data become available.

### 3.4. MB Fpol Correlation with the Tumor Grade

**Figure 5:**
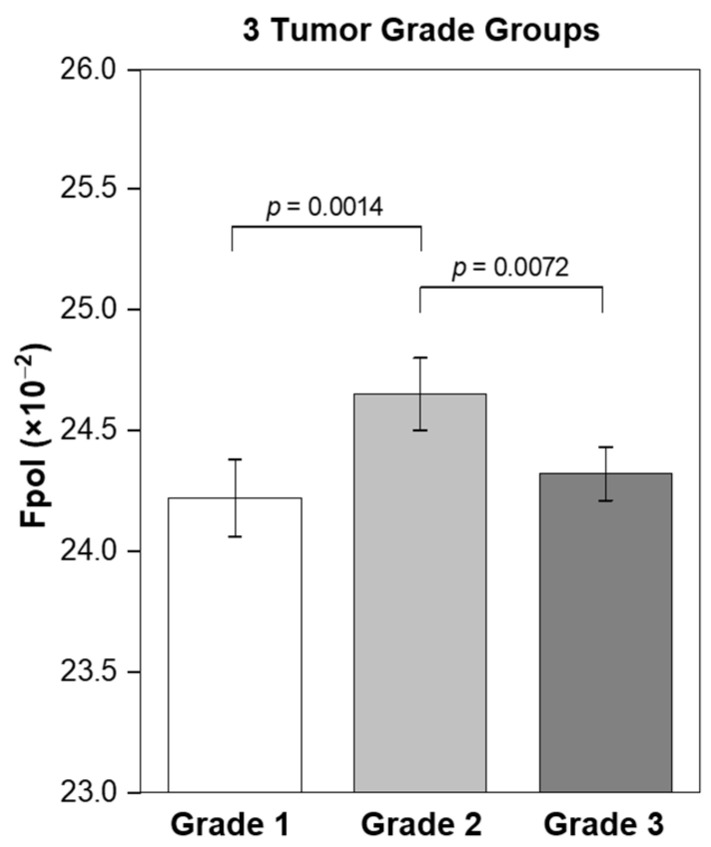
Correlation of MB *Fpol* value with the tumor grade. Grade 1 (2 samples; 185 cells), grade 2 (12 samples; 966 cells), and grade 3 (5 samples; 426 cells). Error bars represent standard errors.

The relationship between tumor grade and *Fpol* values was investigated by comparing optical assessments with routine clinical findings for 19 malignant samples. The IDC and ILC tumors were graded by study pathologists according to standard protocols [33]. Figure 5 shows that tumors with grades 1, 2, and 3 yielded average *Fpol* values of 24.22 ± 0.16 (×10^−2^), 24.65 ± 0.15 (×10^−2^), and 24.32 ± 0.11 (×10^−2^), respectively. Moderately differentiated grade 2 lesions exhibited significantly higher *Fpol* vs. the grade 1 (*p* = 0.0014) or grade 3 (*p* = 0.0072) tumors.

### 3.5. MB Fluorescence Emission Images Display Cytomorphology

**Figure 6:**
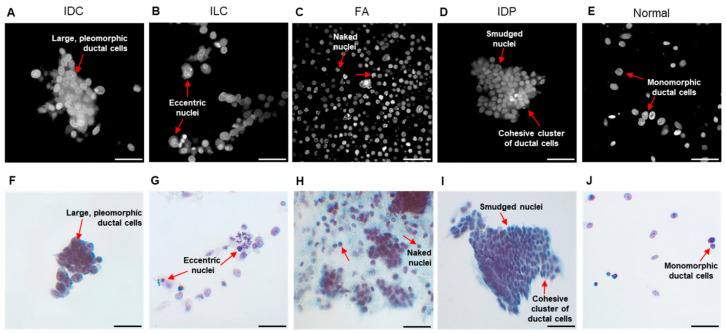
Morphological assessment of representative breast FNA specimens. Example MB fluorescence emission images of cells including (**A**) IDC (subject 2-M), (**B**) ILC (subject 15-M), (**C**) FA (subject 23-B1), (**D**) IDP (subject 27-B), and (**E**) normal (subject 2-N). (**F**–**J**) Corresponding clinical cytology images. Red arrows—cytological features. Scale bar = 50 µm.

*Fpol* images were processed from fluorescence emission images, which could be utilized to provide diagnostic information on cell morphology. For example, Figure 6A–E show fluorescence emission images that correspond to the *Fpol* images in Figure 3A–E. Cytomorphological features of the malignant IDC cells in Figure 6A include overlapping sheets of large pleomorphic ductal cells with prominent nucleoli. In Figure 6B, the fluorescence emission image of cancerous ILC cells shows high cellularity and eccentric nuclei, with cells organized in a linear pattern. Figure 6C,D display representative cells from FA and IDP aspirates, respectively. FA (Figure 6C) exhibits a biphasic population of stromal cells and naked nuclei with clusters of bland epithelial cells. IDP (Figure 6D) shows cellular features including cohesive clusters of ductal cells with smudged nuclei. The normal sample in Figure 6E shows a paucicellular specimen including bland monomorphic ductal cells. Corresponding clinical cytology images display similar features (Figure 6F–J).

## 4. Discussion

Results of our study demonstrated that MB *Fpol* provides accurate differentiation of cancerous vs. noncancerous breast cells and could be used to augment current breast FNA cytology techniques. Statistical analysis revealed significantly increased (*p* < 0.0001) *Fpol* of MB in breast cancer aspirates vs. benign or normal (Figure 2A). Specifically, evaluation by histological grouping revealed *Fpol* values were significantly elevated (*p* < 0.0001) in IDC and ILC samples vs. FA, IDP, and normal samples (Figure 2B). However, there were no significant differences between IDC and ILC specimens. In all the cases, MB *Fpol* assessments of the FNAs correlated with the findings of clinical histopathology. *Fpol* images displayed pronounced contrast between malignant/benign cells (Figure 3A–E), whereas *Fpol* values provided an objective, diagnostic marker for each cell (Figure 3F–J). Therefore, quantitative *Fpol* differences could be used to correctly sort atypical or suspicious lesions (i.e., cases where the differential diagnosis is unclear) into benign or malignant categories.

Notably, there were no cells in benign or normal specimens with an MB *Fpol* value greater than 23.3 (×10^−2^), whereas the *Fpol* values of most cells in malignant samples were similar and significantly higher relative to benign/normal cells (Figure 4). These results indicate that, under well-controlled experimental conditions, there may exist a universal threshold value of MB *Fpol* separating cancerous/noncancerous cells that does not depend on the patient and/or cancer subtype. Moreover, data analysis revealed that even though ~30% of cells in the malignant specimens were noncancerous, an averaged MB *Fpol* value of a cytological sample with about 40–60 cells could serve as a reliable marker for breast cancer. This is an important finding considering that averaged MB *Fpol* could be obtained via spectroscopic measurement and provide a valuable tool for diagnosis in low resource settings, where spatially resolved measurements would be prohibitively expensive.

A significant advantage of MB fluorescence emission and polarization imaging is that it preserved morphology of the cells. Therefore, following optical evaluations, cells could be processed into routine cytological slides. Alternatively, as our results indicate that most cells remained viable after the experiments, MB *Fpol* imaging could be used for in vivo applications. For example, it could guide the collection of FNA specimens or deliver diagnosis in situ, without the need for tissue removal. Further studies are required to explore this prospective application.

Our previous cell culture studies demonstrated that higher *Fpol* values are caused by the shorter fluorescence lifetime of MB in malignant versus normal cells, and the increased uptake of the dye in mitochondria of cancer cells [18,19]. Meanwhile, other groups have reported that positively charged molecules such as MB accumulate in mitochondria due to elevated negative mitochondrial membrane potentials (MMPs) in malignant cells [34,35]. More recently, we showed that MB *Fpol* is increased in clinical aspirates obtained from pathologically diverse, malignant thyroid nodules [21]. As elevated MMPs are a hallmark of both breast and thyroid cancers, the MB *Fpol* method may be capable of detecting additional types of cancer cells with elevated MMPs such as brain, colon, kidney, lung, and pancreatic cancers [19,36,37].

Increased MB uptake in the mitochondria of cancer cells may also increase the fluorescence emission signal. However, the images presented in this manuscript and our previous studies demonstrate that MB localizes to the nuclei, mitochondria, lysosomes, and some other organelles of all cells [18,19,21]. Therefore, it would be challenging to use the fluorescence emission of MB for the reliable detection of cancer. Moreover, considering that fluorescence emission can be strongly modulated by the optical properties of the fluorophore environment, the utilization of the *Fpol* method offers considerable advantages in terms of robustness, reliability, and accuracy.

Statistical analysis revealed correlation between MB *Fpol* values and tumor grades (Figure 5). Grade 2 breast tumors showed significantly higher *Fpol* relative to grade 1 cancers (*p* = 0.0014). Interestingly, the *Fpol* of grade 2 lesions was also significantly elevated as compared to grade 3 (*p* = 0.0072). It has been demonstrated that mitochondrial density is decreased in high grade tumors where metabolic pathways shift from oxidative phosphorylation, which takes place within mitochondria, to glycolysis that occurs in the cytoplasm [37,38,39]. Therefore, as increased *Fpol* is associated with preferential MB uptake in mitochondria [18,19], decreased mitochondrial content and numbers in the grade 3 tumors may provide an explanation for their lower *Fpol* values. The dependence of MB *Fpol* on the tumor grade may prove to be clinically significant, as there may be more subjectivity in defining cytomorphological features, such as scoring nuclear pleomorphism or counting mitoses, in grade 2 vs. grade 1 or grade 3.

## 5. Conclusions

In summary, our results demonstrate that the *Fpol* of the cytological stain MB has significant potential as an accurate, quantitative diagnostic marker for malignancy in clinical breast FNA specimens. Methylene blue *Fpol* can be implemented as a quantitative imaging method for cancer detection in single cells or as a spectroscopic measurement technique, assessing the signal from a collection of cells. It may prove useful as an ancillary technology to reduce the incidence of false negative cytology specimens or implemented as standalone approach. Augmenting conventional cytopathology with an objective quantitative evaluation would provide a minimally invasive, rapid, and cost-efficient method to diagnose and grade breast cancer.

## Figures and Tables

**Table 1:** Characteristics of 44 breast FNA specimens.

Histological Classification	No. of Samples (%)	No. of Cells (%)	Mean *Fpol*, No. ± SE (×10^−2^)
**Malignant**	19 (43)	1577 (41)	24.40 ± 0.17
IDC	15 (34)	1335 (35)	24.42 ± 0.17
ILC	4 (9)	242 (6)	24.24 ± 0.27
**Benign**	10 (23)	910 (24)	19.49 ± 0.23
FA	6 (14)	632 (17)	19.63 ± 0.25
IDP	4 (9)	278 (7)	19.00 ± 0.30
**Normal**	15 (34)	1321 (35)	19.14 ± 0.20

IDC—invasive ductal carcinoma, ILC—invasive lobular carcinoma, FA—fibroadenoma, IDP—intraductal papilloma, SE—standard error.

## Data Availability

The experimental data are available from the corresponding author upon request.

## Supplementary Materials

The following supporting information can be downloaded at: https://www.mdpi.com/article/10.3390/cancers15051501/s1, Table S1: Clinical information and fluorescence polarization (*Fpol*) characteristics of 44breast fine needle aspiration specimens.
