# Supplementary material for: Translational Potential of Fluorescence Polarization for Breast Cancer Cytopathology
Source: Cancers (Basel). 2023 Feb 27;15(5):1501. doi: 10.3390/cancers15051501 (PMC10000687; doi:10.3390/cancers15051501)
Supplement: Supplementary file 1 [file cancers-15-01501-s001.zip › cancers-2201978-supplementary/Cancers_Supplementary Information.pdf]

Supplementary Information for

Translational Potential of Fluorescence Polarization for Breast Cancer Cytopathology

Peter R. Jermain[1,2], Dina H. Kandil[3], Alona Muzikansky[4], Ashraf Khan[5], and Anna N. Yaroslavsky[1,6,*]

[1]Advanced Biophotonics Laboratory, University of Massachusetts Lowell; Lowell, MA 01854, USA.
[2]Department of Radiation Oncology, Massachusetts General Hospital, Boston, MA 02114, USA.
[3]Department of Pathology, UMass Chan Medical School; Worcester, MA 01655, USA.
[4]Biostatistics Center, Massachusetts General Hospital; Boston, MA 02114, USA.
[5]Department of Pathology, UMass Chan Medical School-Baystate; Baystate Health, Springfield, MA 01199, USA.
[6]Department of Dermatology, Massachusetts General Hospital; Boston, MA 02114, USA.
* Correspondence: ayaroslavsky@mgh.harvard.edu; Tel.: 1-978-934-3766

This PDF file includes:

Supplementary Table S1: Clinical information and fluorescence polarization (Fpol) characteristics of 44 breast fine needle aspiration specimens.

Supplementary Table S1. Clinical information and fluorescence polarization (Fpol) characteristics of 44 breast fine needle aspiration specimens.

Subject No.	Age	Tumor dimensions (cm)	Diagnosis, grade	Molecular subtype (ER/PR/HER2)	Surgical treatment	No. of cells analyzed	Mean Fpol ± SD ($\times 10^{-2}$)	Fpol range ($\times 10^{-2}$)	No. of cells with Fpol > 23.3 ($\times 10^{-2}$) (%)
1-M	60	1.2 × 1.0 × 0.5	IDC, 1	ER+/PR+/HER2-	Partial mastectomy	122	24.3 ± 1.7	20.9 – 27.5	91 (75)
2-M	69	0.9 × 0.7 × 0.5	IDC, 2	ER+/PR+/HER2+	Partial mastectomy	211	24.5 ± 1.4	22.0 – 28.5	159 (75)
3-M	46	1.8 × 1.3	IDC, 2	ER+/PR+/HER2-	Partial mastectomy	88	24.7 ± 1.9	22.0 – 28.6	77 (88)
4-M	72	1.1 × 1.0 × 0.6	IDC, DCIS, 2	ER+/PR+/HER2-	Mastectomy	103	24.6 ± 1.3	20.7 – 28.3	92 (89)
5-M1	68	0.9 × 0.8 × 0.6	IDC, DCIS, 2	ER+/PR+/HER2-	Mastectomy	51	24.4 ± 1.6	21.9 – 28.2	33 (65)
5-M2	68	0.9 × 0.8 × 0.6	IDC, DCIS, 2	ER+/PR+/HER2-	Mastectomy	66	24.7 ± 1.8	21.9 – 28.5	49 (74)
6-M	57	2.2 × 1.4	IDC, DCIS, 2	ER+/PR+/HER2-	Partial mastectomy	55	24.5 ± 1.7	20.9 – 29.6	38 (69)
7-M	44	2.2 × 2.0 × 1.5	IDC, DCIS, 2	ER+/PR+/HER2-	Mastectomy	117	24.4 ± 1.4	20.5 – 28.0	85 (73)
8-M	61	1.8 × 1.3 × 0.8	IDC, DCIS, 2	ER+/PR-/HER2-	Partial mastectomy	50	23.8 ± 1.7	21.6 – 29.9	26 (52)
9-M	71	1.5 × 1.0 × 0.7	IDC, DCIS, 2	ER-/PR-/HER2-	Partial mastectomy	46	23.9 ± 1.1	20.1 – 27.8	31 (67)
10-M	61	1.5 × 1.0 × 0.7	IDC, 3	ER+/PR+/HER2-	Partial mastectomy	89	24.5 ± 1.0	22.2 – 27.9	78 (88)
11-M	80	1.6 × 1.6 × 1.2	IDC, DCIS, 3	ER+/PR+/HER2-	Partial mastectomy	103	24.2 ± 1.0	22.0 – 26.8	80 (78)
12-M	69	2.3	IDC, 3	ER-/PR-/HER2+	Partial mastectomy	57	24.1 ± 2.1	17.6 – 29.5	36 (63)
13-M	52	4.2 × 3.5 × 2.8	IDC, DCIS, 3	ER-/PR-/HER2-	Mastectomy	117	23.9 ± 1.6	20.4 – 29.5	69 (59)
14-M	72	0.7 × 0.5 × 0.4	IDC, DCIS, 3	ER-/PR-/HER2-	Partial mastectomy	60	24.9 ± 1.5	22.4 – 29.0	49 (82)
15-M	75	3.0 × 2.7 × 2.0	ILC, LCIS, 1	ER+/PR+/HER2-	Partial mastectomy	63	23.7 ± 1.2	21.4 – 26.5	38 (60)
16-M	69	1.6 × 1.5	ILC, LCIS, 2	ER+/PR+/HER2-	Mastectomy	41	24.0 ± 1.1	20.8 – 31.4	21 (51)
17-M	79	3.0 × 2.7 × 2.0	ILC, LCIS, 2	ER+/PR+/HER2-	Partial mastectomy	62	23.9 ± 1.5	21.2 – 28.0	32 (52)
18-M	60	1.3 × 1.2 × 0.9	ILC, LCIS, 2	ER+/PR+/HER2-	Partial mastectomy	76	24.4 ± 1.8	22.2 – 29.6	64 (84)
19-B	21	4.0	FA	n/a	Partial mastectomy	64	19.8 ± 2.2	15.0 – 23.3	0 (0)
20-B	54	0.6	FA	n/a	Partial mastectomy	117	19.5 ± 1.0	17.5 – 23.0	0 (0)
21-B	25	2.6 × 1.9 × 1.5	FA	n/a	Partial mastectomy	113	19.1 ± 1.7	15.6 – 22.9	0 (0)
22-B	33	10.0	FA	n/a	Partial mastectomy	188	19.2 ± 1.4	16.4 – 22.7	0 (0)
23-B1	20	2.7	FA	n/a	Partial mastectomy	79	19.9 ± 1.3	15.1 – 22.6	0 (0)
23-B2	20	2.6	FA	n/a	Partial mastectomy	71	19.7 ± 0.9	17.4 – 21.7	0 (0)
24-B	87	3.3	IDP	n/a	Partial mastectomy	30	19.0 ± 1.0	16.5 – 20.9	0 (0)
25-B	34	2.3	IDP	n/a	Partial mastectomy	37	18.7 ± 1.3	15.6 – 21.5	0 (0)
26-B	47	1.0	IDP	n/a	Partial mastectomy	97	18.8 ± 1.5	16.2 – 23.0	0 (0)
27-B	54	1.2	IDP	n/a	Partial mastectomy	114	19.1 ± 1.4	15.0 – 23.0	0 (0)
1-N	60	n/a	N	n/a	Partial mastectomy	147	18.1 ± 1.7	15.0 – 21.6	0 (0)
2-N	69	n/a	N	n/a	Partial mastectomy	274	18.7 ± 1.7	14.6 – 22.1	0 (0)
4-N	72	n/a	N	n/a	Mastectomy	47	19.2 ± 1.6	15.6 – 22.1	0 (0)
5-N	68	n/a	N	n/a	Mastectomy	92	19.3 ± 1.3	16.4 – 22.1	0 (0)
6-N	57	n/a	N	n/a	Partial mastectomy	47	20.1 ± 1.7	15.2 – 23.2	0 (0)
7-N	44	n/a	N	n/a	Mastectomy	43	18.8 ± 1.2	16.1 – 21.0	0 (0)
8-N	61	n/a	N	n/a	Partial mastectomy	48	19.9 ± 1.1	17.2 – 22.0	0 (0)
9-N	71	n/a	N	n/a	Partial mastectomy	40	19.6 ± 1.0	18.0 – 21.9	0 (0)
11-N	80	n/a	N	n/a	Partial mastectomy	147	18.4 ± 1.6	14.4 – 21.3	0 (0)
15-N	75	n/a	N	n/a	Partial mastectomy	37	19.7 ± 1.3	18.1 – 21.9	0 (0)
17-N	79	n/a	N	n/a	Partial mastectomy	35	19.8 ± 1.6	14.0 – 21.8	0 (0)
18-N	60	n/a	N	n/a	Partial mastectomy	131	18.9 ± 1.4	15.1 – 22.2	0 (0)
20-N	54	n/a	N	n/a	Partial mastectomy	55	17.6 ± 1.0	15.7 – 20.3	0 (0)
25-N	34	n/a	N	n/a	Partial mastectomy	28	19.3 ± 1.6	16.3 – 21.0	0 (0)
28-N	51	n/a	N	n/a	Partial mastectomy	150	19.5 ± 1.6	15.7 – 23.2	0 (0)

M – malignant, B – benign, N – normal, ER – estrogen receptor, PR – progesterone receptor, HER2 – human epidermal growth factor receptor 2, IDC – invasive ductal carcinoma, ILC – invasive lobular carcinoma, DCIS – ductal carcinoma in situ, LCIS – lobular carcinoma in situ, FA – fibroadenoma, IDP – intraductal papilloma, SD – standard deviation.
